# Clinical and Laboratory Characteristics and Outcome of Illness Caused by Tick-Borne Encephalitis Virus without Central Nervous System Involvement 

**DOI:** 10.3201/eid2802.211661

**Published:** 2022-02

**Authors:** Petra Bogovič, Andrej Kastrin, Stanka Lotrič-Furlan, Katarina Ogrinc, Tatjana Avšič Županc, Miša Korva, Nataša Knap, Franc Strle

**Affiliations:** University Medical Center Ljubljana, Ljubljana, Slovenia (P. Bogovič, S. Lotrič-Furlan, K. Ogrinc, F. Strle);; University of Ljubljana, Faculty of Medicine, Ljubljana (A. Kastrin, T. Avšič Županc, M. Korva, N. Knap)

**Keywords:** tick-borne encephalitis virus, tick-borne encephalitis, TBE, TBEV, central nervous system, febrile illness, thrombocytopenia, leukopenia, viruses, meningitis/encephalitis, vector-borne infections, Slovenia

## Abstract

Information on febrile illness caused by tick-borne encephalitis virus (TBEV) without central nervous system involvement is limited. We characterized 98 patients who had TBEV RNA in their blood but no central nervous system involvement at the time of evaluation. Median duration of illness was 7 days; 37 (38%) patients were hospitalized. The most frequent findings were malaise or fatigue (98%), fever (97%), headache (86%), and myalgias (54%); common laboratory findings were leukopenia (88%), thrombocytopenia (59%), and abnormal liver test results (63%). During the illness, blood leukocyte counts tended to improve, whereas thrombocytopenia and liver enzymes tended to deteriorate. At the time of positive PCR findings, 0/98 patients had serum IgG TBEV and 7 serum IgM TBEV; all patients later seroconverted. Viral RNA load was higher in patients with more severe illness but did not differ substantially in relation to several other factors. Illness progressed to tick-borne encephalitis in 84% of patients within 18 days after defervescence.

## Abstract 2

Illness progressed to encephalitis in 84% of patients within 18 days after defervescence.

Tick-borne encephalitis virus (TBEV) is a member of the genus *Flavivirus* in the family *Flaviviridae* and is transmitted to humans predominantly through *Ixodes* spp. tick bites. In addition to 3 well-known subtypes of TBEV (European, Siberian, and Far Eastern) that cause disease in humans, other subtypes, including the currently named Baikalian and Himalayan subtypes, have been reported (*1*,*2*).

Infection with TBEV can be symptomatic or asymptomatic. As is the case for infections with other flaviviruses, most (70%–98%) persons infected with TBEV do not experience symptoms; however, some findings in blood donors suggest that asymptomatic infections might be rare (*3*). Nevertheless, when infection with TBEV is symptomatic, it can manifest as a febrile illness without central nervous system (CNS) involvement (Figure 1, panel A) but often progresses to tick-borne encephalitis (TBE) (i.e., CNS involvement caused by the virus) (Figure 1, panel B). Clinical manifestation differs in some respects according to virus subtype. In 13%–44% of patients, TBE caused by the European subtype manifests with direct CNS involvement (*4**–**15*) (Figure 1, panel C), whereas in most patients, CNS inflammation is preceded by a febrile illness, resulting in a biphasic course (Figure 1, panel B). The initial phase, which corresponds to viremia, manifests as fever, fatigue, malaise, headache, and muscle and joint pain, but in the absence of CNS inflammation; this phase usually lasts <1 week (*8*), and the illness then improves over a few days. The hallmark of the second phase of the disease is CNS involvement. Meningitis is the predominant manifestation in children. In adults, meningitis occurs in ≈50% of patients, meningoencephalitis in ≈40%, and meningoencephalomyelitis in ≈5%–10%. The case-fatality rate of TBE caused by the European subtype of TBEV is 0.5%–2%. In addition, ≈5% of adult patients are affected by permanent pareses, and at least one third suffer from a postencephalitic syndrome (*16**–**19*).

**Figure 1:**
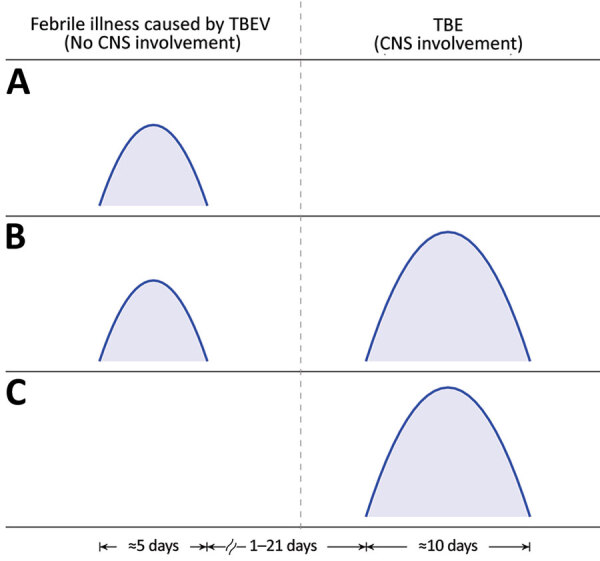
Timelines of clinical manifestations of illness caused by TBEV. A) Febrile headache (fever form); B) biphasic course of TBE; C) monophasic course of TBE. CNS, central nervous system; TBE, tick-borne encephalitis; TBEV, tick-borne encephalitis virus.

In general, clinical manifestations and laboratory characteristics of symptomatic TBEV infection are well described. However, this statement is valid for cases with neurologic involvement (i.e., for TBE) but less so for the initial phase of TBE, and much less so for TBEV infection manifesting solely as febrile illness without later CNS involvement. That manifestation, also called isolated initial phase of TBE, abortive form of TBE, febrile headache, summer flu, or fever form, is postulated to match clinically and serologically the initial phase of TBE, with the exception that subsequent CNS involvement does not occur. TBEV infection manifesting as febrile illness without later CNS involvement is suggested to be frequent (*20**–**23*), although not in all reports (*5*,*6*,*24*,*25*), and the scientific basis for such a conclusion is unclear. Furthermore, although the outcome of symptomatic TBEV infection without CNS involvement is believed to be favorable, no reliable data on the outcome have been published. Because clinical symptoms and signs of the illness are nonspecific, and because, in parallel to the initial phase of TBE, serum antibodies to TBEV are not yet expected to have developed, the only option for diagnosis at the time of actual illness is demonstrating the presence of TBEV RNA in the blood. However, this approach is not routine and might have a low diagnostic yield owing to several other known or unknown causes of fever, even in a region that is highly endemic for TBE. Therefore, the possibility that a febrile illness is the result of TBEV infection is usually tested for and established only after signs or symptoms of CNS involvement appear, which does not happen in case of the fever form. In that case (and if PCR detection of viral RNA in blood is not available), further clinical and microbiologic (serologic) follow-up after improvement is needed to establish the diagnosis. In this study, we analyzed in detail the clinical and laboratory characteristics of febrile illness after tick bite or exposure to ticks and its outcome in patients in whom infection with TBEV was established by the presence of viral RNA in the blood.

## Materials and Methods

### Definitions

Febrile illness resulting from infection with TBEV was defined by the presence of fever and constitutional symptoms, demonstration of viral RNA in serum specimens, and the absence of signs or symptoms of CNS involvement at the time of illness. In patients with clinical signs potentially suggesting CNS involvement, cerebrospinal fluid (CSF) samples were examined; the threshold for lumbar puncture was low. A CSF leukocyte count <5 × 10^6^/L was interpreted as excluding CNS inflammation.

According to the later appearance (or absence) of neurologic involvement, the febrile illness was further subclassified as either the initial phase of TBE (defined as a febrile illness with demonstration of viral RNA in serum samples that, after a clinical improvement, was followed by neurologic involvement within a 2-month follow-up period and fulfilling criteria for TBE) or as febrile illness resulting from infection with TBEV in a narrow sense (fever form, febrile headache) when no signs of CNS involvement were present at the time of actual illness or within a 2-month follow-up period. TBE was defined as the presence of clinical signs or symptoms of meningitis or meningoencephalitis, increased CSF leukocyte counts (>5 × 10^6^ cells/L), and demonstration of a recent infection with TBEV indicated by serum IgM and IgG or IgG seroconversion in paired serum samples.

### Patients and Samples

Adult patients examined for febrile illness at the Department of Infectious Diseases, University Medical Center Ljubljana (Ljubljana, Slovenia), during 2003–2019, in whom the presence of TBEV RNA was identified by PCR in serum specimens, qualified for the study. Serum samples were obtained either during a prospective study on the etiology of febrile illness after a tick bite or exposure to ticks (62 patients, 63.3%) or represented remnants of samples collected as a part of routine diagnostic testing of patients with febrile illness in whom TBE later occurred (36 patients, 36.7%). Serum specimens were stored at –80°C until further processing. For the 62 patients, we obtained clinical and laboratory information on the etiology of febrile illness occurring after tick bite or tick exposure prospectively. Clinical and laboratory follow-up occurred for these patients for at least 2 months (i.e., at first evaluation and at follow-up visits 1 week, 2 weeks, and 2 months later). For the other 36 patients, we obtained clinical and laboratory information from medical charts.

### TBEV Antibodies and RNA Load

We determined the presence of TBEV antibodies in serum samples by using the Enzygnost Anti-TBE/FSME Virus (IgM, IgG) test (Siemens AG, https://www.siemens.com), according to the manufacturer’s instructions. We extracted total RNA from serum samples by using the QIAamp Viral RNA Mini Kit (QIAGEN, https://www.qiagen.com), according to the manufacturer’s instructions. For the detection of TBEV RNA, we performed quantitative reverse transcription PCR as reported previously (*26*).

### Statistical Analysis

We summarized continuous variables as median values and interquartile ranges (IQRs), and discrete variables as counts and percentages with 95% CIs. We based comparisons between groups on Wilcoxon rank-sum tests for continuous variables and Fisher exact tests for discrete variables. We defined statistical significance as a p value of <0.05.

**Figure 2:**
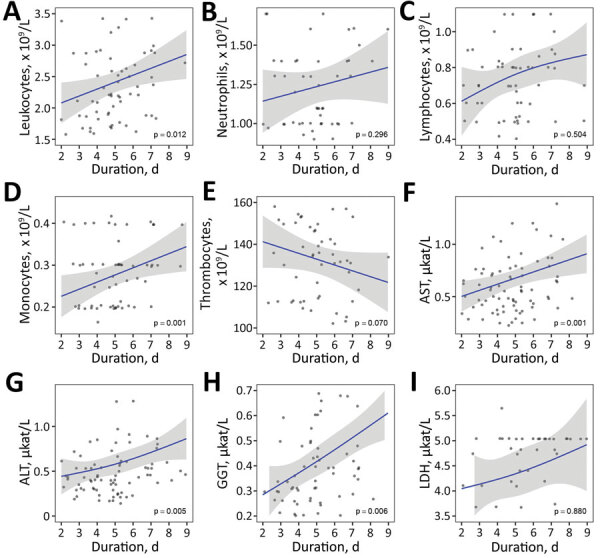
Laboratory findings according to illness duration in cases of febrile illness caused by tick-borne encephalitis virus without central nervous system involvement at the time of evaluations, Slovenia. A) Leukocytes, B) neutrophils, C) lymphocytes, D) monocytes, E) thrombocytes, F) AST, G) ALT, H) GGT, and I) LDH. Blue lines indicate loess regression lines; shaded areas indicate 95% CIs. Relationships between variables in panels C, G, and I were modeled by using restricted cubic splines with 3 knots (*25*). ALT, alanine aminotransferase; AST, aspartate aminotransferase; GGT, gamma-glutamyl transferase; LDH, lactate dehydrogenase.

**Figure 3:**
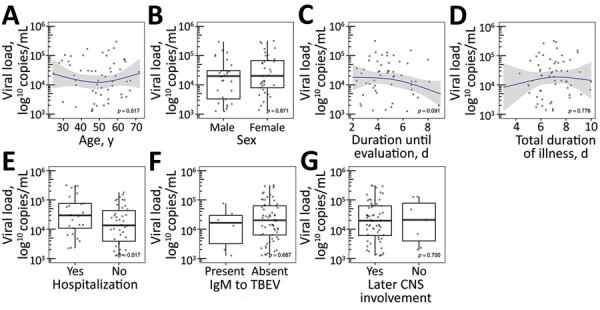
TBEV RNA load according to demographic and clinical characteristics in cases of febrile illness without central nervous system involvement at the time of evaluations, Slovenia. A) Age, B) sex, C) duration of illness until evaluation, D) total duration of illness, E) hospitalization, F) presence of IgM to TBEV, and G) later CNS involvement. Blue lines indicate loess regression lines; shaded areas indicate 95% CIs. Relationships between variables in panels A, B, and D are modeled by using restricted cubic splines with 3 knots (*25*). Comparisons between groups in panels B, E, F, and G are based on a Wilcoxon rank-sum test. CNS, central nervous system; TBEV, tick-borne encephalitis virus.

We examined associations between variables by using linear regression modeling (Figures 2, 3). We used log_10_-transformed viral RNA loads, and domain experts (P.B. and F.S.) selected included covariates. We modeled continuous covariates that demonstrated a nonlinear relationship by using restricted cubic splines (*27*) and imputed missing values by using multiple imputation on the basis of additive regression, bootstrapping, and predictive mean matching (*28*). We used R software for all statistical analyses (*29*).

### Ethics

The study was conducted in accordance with the principles of the Declaration of Helsinki, the Oviedo Convention on Human Rights and Biomedicine, and the Slovene Code of Medical Deontology. The study was approved by the National Medical Ethics Committee of Slovenia (approval nos. 152/06/13, 178/02/13, and 37/12/13). Patients whose specimens were obtained in the study on the etiology of febrile illness after a tick bite or exposure to ticks signed an informed consent form. The Ethics Committee waived the need for written informed consent for patients for whom remnants of routinely collected serum specimens were used.

## Results

A total of 98 adult patients examined for febrile illness in whom TBEV RNA was identified by PCR in their serum specimens were enrolled in the study. The median age of the patients was 51 years; 52% were women.

### Clinical and Laboratory Characteristics of Febrile Illness Caused by TBEV

Most (88.7%) patients reported a tick bite within 4 weeks of the onset of illness. The median time from the bite to illness onset was 6 days, median duration of illness before evaluation was 5 days, and total duration of the illness was 7 days. A total of 37/98 (37.8%) patients were hospitalized for a median of 3 days. The most frequent symptoms or signs were malaise and fatigue (98%), fever (96.9%), headache (85.7%), and myalgias (54.1%) (Table 1). The most frequent laboratory findings were leukopenia (87.5%), thrombocytopenia (59.4%), and abnormal liver test results (62.5% of patients had >1 abnormal liver test result, most often elevated aspartate aminotransferase [AST, 55.0%] and alanine aminotransferase [ALT, 26.3%]) (Table 2).

**Table 1:** Demographic and clinical data on adult patients who had febrile illness caused by tick-borne encephalitis virus without central nervous system involvement at the time of evaluation, Slovenia*

Characteristic	Value
Sex	
F	51 (52.0, 41.7–62.2)
M	47 (48.0, 37.8–58.3)
Median age, y (IQR)	51 (37–60.75)
Underlying illnesses	38 (38.8, 29.1–49.2)
History of tick bite†	86 (88.7, 80.6–94.2)
Median incubation period, d (IQR)‡	6 (4–9.75)
Median duration of illness before first evaluation, d (IQR)	5 (4–6)
Clinical manifestation	
Body temperature >37.5°C	95 (96.9, 91.3–99.4)
Median body temperature, °C (IQR)§	38.3 (37.8–38.9)
Chills	19 (19.4, 12.1–28.6)
Headache	84 (85.7, 77.2–92.0)
Myalgia	53 (54.1, 43.7–64.2)
Arthralgia	42 (42.9, 32.9–53.3)
Gastrointestinal symptoms	45 (45.9, 35.8–56.3)
Abdominal pain	2 (2.0, 0.3–7.2)
Nausea, vomiting	37 (37.8, 28.2–48.1)
Diarrhea	16 (16.3, 9.6–25.2)
Malaise and fatigue	96 (98.0, 92.8–99.8)
Respiratory symptoms	18 (18.4, 11.3–27.5)
Sore throat	11 (11.2, 5.7–19.2)
Cough	10 (10.2, 5.0–18.0)
Median duration of illness, initial phase, d (IQR)¶	7 (6–8)
Hospitalization	
No. hospitalized patients	37 (37.8, 28.2–48.1)
Median duration of hospitalization, d (IQR)#	3 (1–5)

*Values are no. (%, 95% CI) except as indicated. IQR, interquartile range. †Tick bite within 4 weeks before onset of illness. Data available for 97 patients. ‡Time (days) from tick bite to onset of illness calculated only in patients with 1 bite. Data available for 54 patients. §Highest temperature in the course of the illness. ¶Data available for 82 patients. #Data available for 33 patients.

**Table 2:** Laboratory data on adult patients who had febrile illness caused by tick-borne encephalitis virus without central nervous system involvement at the time of evaluation, Slovenia*

Laboratory findings	Value	
Median blood leukocyte count, × 10^9^/L (IQR)†	2.3 (1.8–3.125)	
Blood leukocyte count <4 × 10^9^/L	84 (87.5, 79.2–93.4)	
Blood leukocyte count >10 × 10^9^/L	0 (0, 0–3.8)	
Median blood neutrophil count, × 10^9^/L (IQR)‡	1.22 (0.9–1.7)	
Blood neutrophil count <1.5 × 10^9^/L	55 (65.5, 54.3–75.5)	
Blood neutrophil count >7.4 × 10^9^/L	0 (0, 0–4.3)	
Median blood lymphocyte count, × 10^9^/L (IQR)‡	0.88 (0.5–1.1)	
Blood lymphocyte count <1.1 × 10^9^/L	61 (72.6, 61.8–81.8)	
Blood lymphocyte count >3.5 × 10^9^/L	1 (1.2, 0–6.5)	
Median blood monocyte count, × 10^9^/L (IQR)‡	0.29 (0.2–0.4)	
Blood monocyte count <0.21 × 10^9^/L	34 (40.5, 29.9–51.8)	
Blood monocyte count >0.92 × 10^9^/L	0 (0, 0–4.3)	
Median blood platelet count, × 10^9^/L (IQR)†	132 (110.5–157)	
Blood platelet count <140 × 10^9^/L	57 (59.4, 48.9–69.3)	
Serum C-reactive protein level >5 mg/L†§	9 (9.4, 4.4–17.1)	
Liver test results, µkat/L (IQR)¶		
Median alkaline phosphatase¶	0.93 (0.755–1.06)	
Elevated >1.92	0 (0, 0–4.6)	
Median aspartate aminotransferase#	0.615 (0.4575–0.8325)	
Elevated >0.58	44 (55.0, 43.5–66.2)	
Median alanine aminotransferase#	0.53 (0.37–0.805)	
Elevated >0.77	21 (26.3, 17.0–37.3)	
Median gamma-glutamyl transferase¶	0.37 (0.265–0.595)	
Elevated >0.92	9 (11.4, 5.3–20.5)	
Median lactate dehydrogenase**	3.48 (2.92–4.82)	
Elevated >4.13	12 (36.4, 20.4–54.9)	
No. patients with >1 abnormal function test result††	50 (62.5, 51.0–73.1)	

*Values are no. (%, 95% CI) unless otherwise noted. IQR, interquartile range. †Data available for 96 patients. ‡Data available for 84 patients. §Median 7, range 6–45. ¶Data available for 79 patients. #Data available for 80 patients. **Data available for 33 patients. ††Data available for 80 patients.

Individual laboratory parameters according to duration of illness before testing demonstrated heterogeneous results. Counts of total peripheral blood leukocytes, neutrophils, lymphocytes, and monocytes were lowest early in the course of illness and tended to increase (improve) with the duration of illness, whereas thrombocytopenia became more pronounced with the duration of illness. Liver tests, including AST, ALT, gamma-glutamyl transferase (GGT), and lactate dehydrogenase, also tended to deteriorate with the duration of illness. These tendencies were noticeably uniform and were significant for total leukocyte and monocyte counts and for AST, ALT, and GGT levels (Figure 2).

None of the 98 patients had serum IgG to TBEV at the time of a positive PCR result (median of 5 days after illness onset) and only 2/98 (2.0%) had TBEV-specific IgM. In these 2 patients, the serologic tests were performed on days 7 and 13 of the illness. An additional 5 patients (5.1%) had borderline specific IgM levels; for most, the duration of illness before testing was somewhat longer (median 6 days, range 3–9 days). Viral RNA load was higher in hospitalized patients with more severe illness than in those who did not need hospitalization but did not differ substantially according to age, sex, duration of illness before testing, or total duration of the actual febrile illness, or for patients with undetectable viral IgM in serum samples when compared with patients in whom antibodies were detectable (Figure 3).

### Outcome

Of the 62 patients who received a diagnosis during a prospective study on the etiology of febrile illness after a tick bite or exposure to ticks and in whom TBEV was present in serum samples, 6 (9.7%) did not experience any symptoms or signs during the follow-up period of 2 months. In 4 (4.5%) patients, mild constitutional symptoms not suggesting CNS involvement and without meningeal signs reappeared after a symptom-free interval of up to 12 days. In contrast, the other 52 (83.9%) patients experienced overt signs of meningitis or meningoencephalitis associated with CSF pleocytosis and fulfilled serologic criteria for TBE; in this subgroup, the longest symptom-free interval was 18 days.

The clinical characteristics of the initial phase of TBE and febrile illness without subsequent CNS involvement were not formally compared because the number of patients was too small, but the clinical and laboratory manifestation of the 2 entities appears comparable (Table 3). All patients developed IgM and IgG to the virus during follow-up.

**Table 3:** Basic demographic, clinical, and laboratory characteristics of febrile illness caused by tick-borne encephalitis virus infection with or without subsequent central nervous system involvement*

Characteristic	Febrile illness followed by CNS involvement, n = 52		Febrile illness with possible later CNS involvement, n = 4		Febrile illness without later CNS involvement, n = 6	
Sex						
F	26 (50.0, 35.8–64.2)		2 (50.0, 6.8–93.2)		3 (50.0, 11.8–88.2)	
M	26 (50.0, 35.8–64.2)		2 (50.0, 6.8–93.2)		3 (50.0, 11.8–88.2)	
Median age, y (IQR)	50 (40.75–62)		52 (51.75–55)		45.5 (34.5–55)	
Underlying illnesses	22 (42.3, 28.7–56.8)		0 (0, 0–60.2)		3 (50.0, 11.8–88.2)	
History of tick bite†	45/51 (88.2, 76.1–95.6)		3/4 (75.0, 19.4–99.4)		5/6 (83.3, 35.9–99.6)	
Median incubation period, d (IQR)‡	6 (4.5–9.5)§		9 (9–9)¶		6.5 (3–11)#	
Median duration of illness before first evaluation, d (IQR)	5 (3.75–7)		5.5 (5–6.25)		5 (4.25–6.5)	
Clinical manifestation						
Body temperature >37.5°C	51/52 (98.1, 89.7–100)		3/4 (75.0, 19.4–99.4)		6 (100, 54.1–100)	
Median body temperature, °C (IQR)**	38.5 (38–39)		38.3 (38.05–38.45)		39 (38.625–39)	
Chills	10 (19.2, 9.6–32.5)		1 (25.0, 0.6–80.7)		1 (16.7, 0.4–64.1)	
Headache	47 (90.4, 79.0–96.8)		2 (50.0, 6.8–93.2)		6 (100, 54.1–100)	
Myalgias	27 (51.9, 37.6–66.0)		3 (75.0, 19.4–99.4)		3 (50.0, 11.8–88.2)	
Arthralgias	22 (42.3, 28.7–56.8)		3 (75.0, 19.4–99.4)		2 (33.3, 4.3–77.7)	
Gastrointestinal symptoms	24 (46.2, 32.2–60.5)		2 (50.0, 6.8–93.2)		3 (50.0, 11.8–88.2)	
Abdominal pain	1 (1.9, 0.1–10.3)		1 (25.0, 0.6–80.7)		0 (0, 0–45.9)	
Nausea, vomiting	22 (42.3, 28.7–56.8)		2 (25.0, 0.6–80.7)		2 (33.3, 4.3–77.7)	
Diarrhea	6 (11.5, 4.4–23.4)		2 (50.0, 6.8–93.2)		1 (16.7, 0.4–64.1)	
Malaise or fatigue	52 (100, 93.2–100)		4 (100, 39.8–100)		5 (83.3, 35.9–99.6)	
Respiratory symptoms	7 (13.4, 5.6–25.8)		0 (0, 0–60.2)		1 (16.7, 0.4–64.1)	
Sore throat	7 (13.4, 5.6–25.8)		0 (0, 0–60.2)		1 (16.7, 0.4–64.1)	
Cough	1 (1.9, 0.1–10.3)		0 (0, 0–60.2)		0 (0, 0–45.9)	
Median duration of illness, d (IQR)	7 (6–8)††		8 (7–9)‡‡		9 (7.5–9.75)	
Hospitalization						
No. hospitalized patients	20 (38.5, 25.3–53.0)		2 (50.0, 6.8–93.2)		3 (50.0, 11.8–88.2)	
Median duration, d (IQR)	4 (1–6)§§		4.5 (3.75–5.25)		4 (2.5–5)	
Laboratory findings						
Median blood leukocyte count, × 10^9^/L (IQR)	2.2 (1.8–2.85)¶¶		1.7 (1.675–1.95)		2.95 (2.5–4.15)	
Blood leukocyte count <4 × 10^9^/L	50 (100, 92.9–100)		4 (100, 39.8–100)		4 (66.7, 22.3–95.7)	
Blood leukocyte count >10 × 10^9^/L	0 (0, 0–7.1)		0 (0, 0–60.2)		0 (0, 0–45.9)	
Median blood platelet count, × 10^9^/L (IQR)	125.5 (108.25–152.75)¶¶		110 (86–134.25)		139 (118.5–162.5)	
Blood platelet count <140 × 10^9^/L	34 (68.0, 53.3–80.5)		3 (75.0, 19.4–99.4)		3 (50.0, 11.8–88.2)	
Serum C-reactive protein elevated, >5 mg/L	3/50 (6.0, 1.3–16.6)##		0 (0, 0–60.2)		1 (16.7, 0.4–64.1)***	
Liver tests, µkat/L (IQR)						
Median aspartate aminotransferase	0.605 (0.4575–0.84)†††		1.05 (0.59–3.55)		0.62 (0.5075–0.845)	
Elevated >0.58	26 (54.2, 39.2–68.6)		3 (75.0, 19.4–99.4)		4 (66.7, 22.3–95.7)	
Median alanine aminotransferase	0.535 (0.4–0.82)†††		1.285 (0.48–3.5225)		0.56 (0.3475–0.78)	
Elevated >0.77 µkat/L	13 (27.1, 15.3–41.9)		2 (50.0, 6.8–93.2)		2 (33.3, 4.3–77.7)	
No. patients with >1 abnormal liver test result	29/48 (60.4, 45.3–74.2)		3 (75.0, 19.4–99.4)		4 (66.7, 22.3–95.7)	

*Values are no. (%, 95% CI) except as indicated. CNS, central nervous system; IQR, interquartile range. †Tick bite within 4 weeks before onset of illness. ‡Time (d) from tick bite to onset of illness calculated only in patients with 1 bite. §Data available for 27 patients. ¶Data available for 1 patient. #Data available for 4 patients. **The highest temperature in the course of the illness. ††Data available for 45 patients. ‡‡Data available for 2 patients. §§Data available for 17 patients. ¶¶Data available for 50 patients. ##Median 6, range 6–9. ***Value 45. †††Data available for 48 patients.

Of the 62 prospectively followed patients, 27 (43.5%) were hospitalized. The likelihood of later CNS involvement in hospitalized patients was similar to that in patients with less severe illness who were treated as outpatients (24/27 [88.9%] vs. 28/35 [80%]; p = 0.49). Furthermore, the level of viral RNA in serum samples in the group in which no CNS involvement occurred was similar to the level in those in whom meningitis or meningoencephalitis later occurred (Figure 3).

## Discussion

Symptomatic infection with TBEV can manifest as CNS involvement (TBE) or as a febrile illness with or without subsequent CNS involvement (Figure 1). Although the epidemiology and clinical manifestation of symptomatic TBEV infection is considered well-established, this statement is valid only for neurologic involvement (TBE) and is less well-established for the initial phase of TBE and even less so for TBEV infection manifesting solely as febrile illness without later CNS involvement.

TBE is highly endemic to Slovenia. For as many as 70% of patients in Slovenia who have notified cases of TBE, diagnosis occurs at University Medical Center Ljubljana (*30*,*31*), which provided access to detailed information on a large number of patients with TBE and enabled this study.

The initial phase of TBE consists of fever, headache, myalgias, arthralgias, and fatigue (*5*,*6*,*8*,*9*,*13*,*32**–**34*), but reliable information on the relative frequency of individual symptoms is limited, and results are variable. In this study, which encompasses TBEV febrile illness with and without later CNS involvement (the initial phase of TBE and febrile headache) and is based on a well-defined group of patients with definite proof of TBEV infection (the presence of virus in blood at the time of actual illness and later seroconversion), our findings corroborate previous results on the spectrum of symptom manifestation and add reliable information on their relative frequency. Thus, >85% of patients have malaise or fatigue, fever, and headache; ≈50% of the patients report myalgias, arthralgias, and, rather unexpectedly, gastrointestinal symptoms (abdominal pain, nausea, vomiting, or diarrhea); and the frequency of respiratory symptoms or chills is almost 20%. The finding of chills is somewhat surprising because chills are not common in patients with viral infections and are more characteristic of diseases caused by bacteria. Illness duration in our patients (median 7 days) was somewhat longer than reported in the literature (median 4–6, range 1–19 days) (*5*,*6*,*8*,*9*,*12*,*26*,*32*,*34*). In addition, in some patients, the illness was relatively severe: more than one third of patients were hospitalized. Laboratory findings possibly contributed to decisions to hospitalize, because leukopenia and thrombocytopenia might suggest serious disease in a patient with fever.

The first phase of TBE is known to be accompanied by leukopenia; thrombocytopenia and abnormal liver test results also might be present, although to a lesser extent (*35*,*36*). In contrast, in the second phase of TBE, the blood leukocyte count is elevated or in normal range. Laboratory tests in the patients in our study very often demonstrated abnormalities (leukopenia in 88%, abnormal liver tests in 70%, and thrombocytopenia in 59%) that were more common than previously described (*6*,*13*,*25*,*26*,*35*,*36*). Our study corroborates previous findings that in most patients, the concentration of total leukocytes in the peripheral blood is reduced. In addition, we offer several new findings, such as a reduction in all major subgroups of leukocytes and a tendency for total numbers of leukocytes, neutrophils, lymphocytes, and monocytes to increase (i.e., improve). In contrast, thrombocytopenia, liver tests (including AST, ALT, and GGT), and lactate dehydrogenase tended to deteriorate with duration of illness. Although we do not have an exact explanation for these laboratory abnormalities, they seem biologically plausible. During illness caused by TBEV without CNS involvement (including illness with subsequent CNS involvement), TBEV replicates in various organs and tissues, and this might affect test results. Later, however, when viremia vanishes and CNS damage occurs, the abnormalities are not present (*7*,*8*,*16**–**18*) and obviously are temporally associated with viremia, suggesting a direct or indirect effect of the virus on the bone marrow and liver.

Our results corroborate previous findings (*24*,*26*,*37*) that the appearance of antibodies to TBEV greatly diminishes the likelihood of detecting viral RNA in blood: none of our 98 patients had detectable serum IgG to TBEV at the time of a positive PCR finding, although 7 (7.6%) patients had specific IgM. As expected, in these 7 patients, the duration of illness was longer than for patients who were completely seronegative. We also expected that the viral RNA load would be lower in patients with detectable serum IgM to the virus than in patients in whom the antibodies were undetectable, but we did not confirm this premise. Furthermore, viral RNA load did not differ substantially in relation to age, sex, duration of illness before testing, or total duration of the actual febrile illness; however, viral RNA load was higher in patients with more severe illness (those who were hospitalized compared with those who were not). Information on TBEV RNA load in humans is very limited (*24*,*26*). In our previous report on viral RNA load in patients with biphasic course of TBE, the load was higher in women than in men but was not significantly associated with clinical and laboratory characteristics of the initial phase of illness or with characteristics of the later meningoencephalitic phase of TBE (*26*). However, because several associations tested in this study were not analyzed in our previous study (and vice versa), direct comparison is restricted to matching approaches; within them, the only discordant result was for viral RNA load according to sex, which was significantly higher in women than men in the previous study but not in this study.

Some review articles have stated that febrile illness without later CNS involvement is a frequent clinical manifestation of infection with TBEV, representing approximately two thirds of all clinically manifested infections with TBEV (*20**–**23*). However, a PubMed literature search of titles and abstracts using the search terms “(tick-borne encephalitis) AND (initial phase OR first phase)” and without time limitation (i.e., from 1966 onward) did not reveal any primary source firmly supporting such statements for Western and Central Europe. Nevertheless, febrile illness resulting from TBEV infection without subsequent CNS involvement is usually not recognized, possibly because of the small proportion of such cases (*38*), which is in accordance with some other reports (*5*,*6*,*24*,*25*). Our previous findings, which were based on clinical and serologic analyses of febrile illness after a tick bite, suggested that febrile illness resulting from infection with TBEV occurs as a rule with subsequent CNS involvement (TBE) after an improvement of up to 12 days (*25*,*34*). In this study, which was focused on 62 patients with well-defined TBEV infection, 52 (84%) patients experienced overt symptoms and signs of CNS involvement associated with CSF lymphocytic pleocytosis, 6 (10%) patients remained completely asymptomatic, and 4 (6%) patients in whom CSF was not examined experienced mild symptoms not associated with meningeal signs for a duration of 2–7 days. We expected that patients in whom TBE did not occur would have lower blood levels of virus than those in whom CNS involvement occurred; these speculations were not confirmed.

The main strengths of our study are the sufficiently large number of patients with a well-defined diagnosis of febrile illness resulting from TBEV infection (on the basis of demonstration of viral RNA in serum samples during actual illness and on later seroconversion), together with the comprehensive collection of clinical and laboratory data and assessment of the course and outcome of the illness. We reveal several new clinical and laboratory details of febrile illness caused by TBEV, confirming that in most (at least 84%) of these patients, TBE (i.e., CNS inflammation) later develops. However, because we included only patients referred to us by family physicians, one of the limitations of the study is a potential selection bias: patients with very mild illness probably do not visit primary physicians and, even if they do, the likelihood of referral to us is lower than for patients with more severe disease or unusual laboratory findings, such as leukopenia or thrombocytopenia. Thus, our findings are limited to a subset of patients with more severe disease; consequently, our conclusions might not be valid for milder clinical cases.

In conclusion, febrile illness caused by TBEV infection is characterized clinically by the presence of malaise or fatigue (98%), fever (97%), headache (86%), and myalgias (54%) and in laboratory tests by leukopenia (88%), thrombocytopenia (59%), and abnormal liver results (63% of patients had >1 abnormal liver test, usually elevated AST and ALT) but normal inflammatory markers. The infection proceeded to TBE in >5/6 (84%) patients within 18 days after defervescence. Clinical and laboratory findings in patients with TBEV febrile illness do not distinguish between patients in whom TBE later develops and those in whom it does not.
